# Effectiveness of a social-emotional learning program on developmental assets and subjective well-being

**DOI:** 10.1038/s41598-023-42040-1

**Published:** 2023-09-12

**Authors:** Rabea Aghatabay, Seyed Saeed Mazloomy Mahmoodabad, Aliakbar Vaezi, Mehdi Rahimi, Hosein Fallahzadeh, Somayeh Alizadeh

**Affiliations:** 1 Department of Health Education and Promotion, School of Public Health, Shahid Sadoughi University of Medical Sciences and Health Services, Yazd, Iran; 2 Department of Public Health, Shahid Sadoughi University of Medical Sciences and Health Services, Yazd, Iran; 3 Department of Nursing, Shahid Sadoughi University of Medical Sciences and Health Services, Yazd, Iran; 4 Department of Psychology and Education, Yazd University, Yazd, Iran; 5 Department of Epidemiology and Biostatistics, Shahid Sadoughi University of Medical Sciences and Health Services, Yazd, Iran; 6 Department of Health Education and Promotion, School of Public Health, Kerman University of Medical Sciences, Kerman, Iran

**Keywords:** Psychology, Health care

## Abstract

The present study was done to evaluate the effectiveness of a Social Emotional Learning (SEL) intervention designed based on social marketing on developmental assets and the well-being of female adolescents in Yazd, Iran. This mixed-method quasi-experimental study was conducted in 2018–2019. A total of 190 female students were selected by multi-stage sampling from the female middle and high schools in Yazd, Iran. A SEL intervention designed based on social marketing principles was implemented among the parents and students of the intervention group. The control group did not receive any intervention. Quantitative data were collected in three stages: pre-test, post-test after two months, and follow-up after six months via Developmental Assets Profile and EPOCH measure of wellbeing. A qualitative evaluation was also performed after the intervention. Results of the Repeated-Measures test indicated that the intervention significantly influenced social competence (p-value = .02). However results did not show improvement in the positive identity development of the participant girls. Qualitative findings suggested the effectiveness of the intervention on social competencies and positive identity. Based on the results of the present study, the SEL program might have a significant but small positive effect on the social competencies of the participating adolescents.

## Introduction

**Figure 1:**
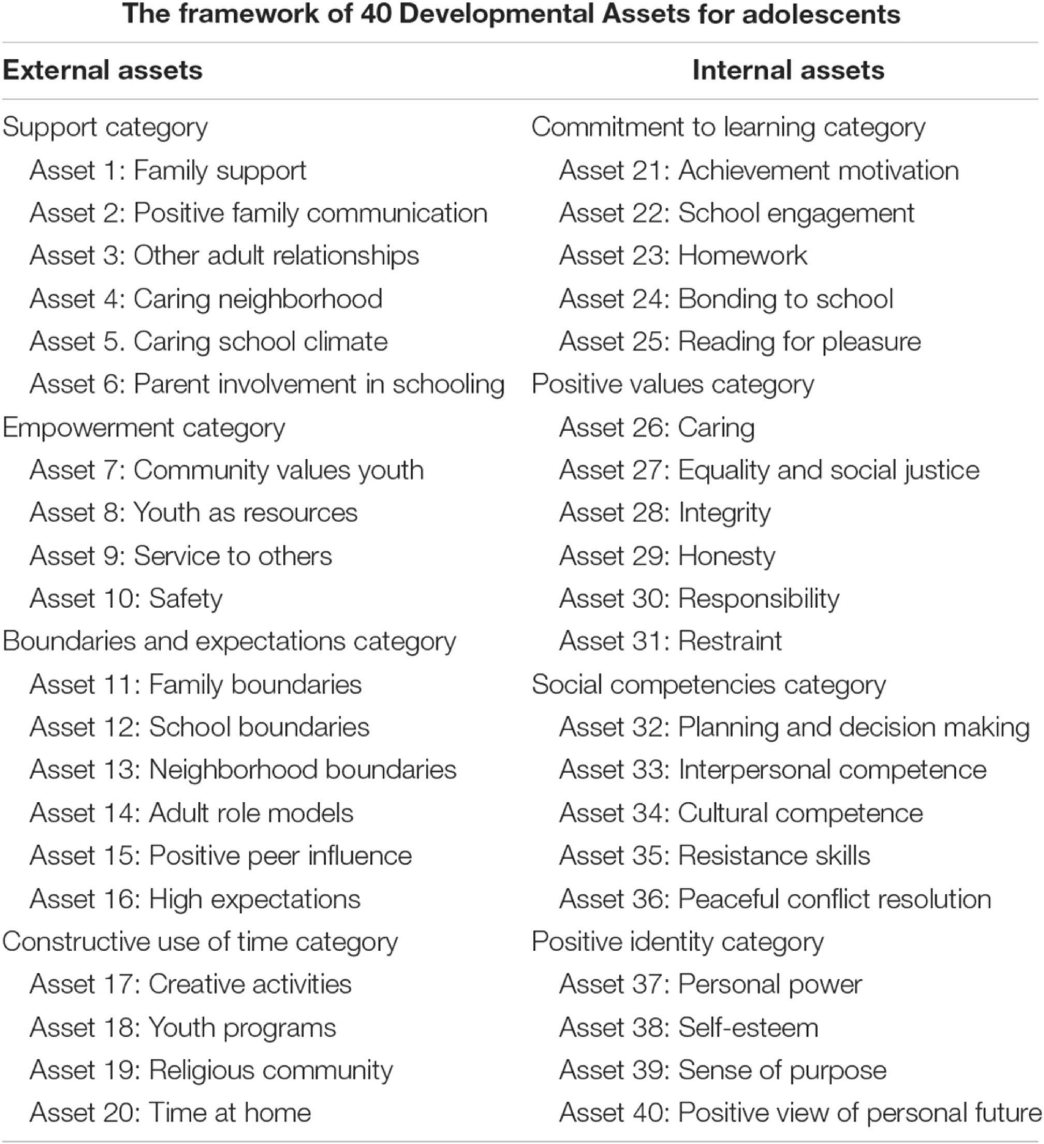
Developmental assets of Search Institute.

Researchers in the field of adolescent’s well-being have recently concluded that Positive Youth Development (PYD) can be the golden key to promote adolescent’s health and prevent risky behaviors in them^1,2^. According to PYD, adolescents' strengths and skills along with contextual resources can lead to development of thriving, healthy, happy, and resilient adolescents^3^. The Developmental Assets Framework is one of the most prominent efforts made to extend PYD both theoretically and practically in the world, according to which, it has been attempted to encompass all the necessary resources for PYD, such as individual, family, social, school, and community assets^4,5^. Results of several studies have confirmed that level of developmental assets is associated with healthy and risky behaviors among adolescents^6–8^. There are 40 developmental assets which has been shown to be critical for adolescent’s health and wellbeing. Figure 1 display all of the 40 developmental assets in their categories which have been introduced by Search Institute (1997).

PYD results from alignment of the adolescent’s skills and strengths with contextual resources. A challenge in designing PYD-promoting interventions is that different contexts offer different levels of resources for adolescents^9^. Emerging requirements of the world in twenty-first century where the adolescents grow up in increasingly dynamic and heterogeneous societies make it even more challenging^10^. In addition, according to Learner et al., adolescents are conscious agents who influence their own development trying to make their personal way in a complex world. They play a creative role in arranging complex puzzle of their developmental tasks^2^. Therefore, recognizing different characteristics and needs of the adolescents living in variant communities is necessary to create effective programs in order to promote their positive development^11^.

Therefore, the present study was conducted to promote well-being and developmental assets of female adolescents in Yazd (Iran). CDCynergy social marketing software was used to plan the study and design interventions consistent with the context, characteristics, culture, and needs of Iranian adolescent girls participated in this study.

CDCynergy is a methodology tool used for health communication planning and implementation; a social marketing edition of CDCynergy was introduced in 2005 by the Centre for Disease Control (CDC). CDCynergy social marketing planning model was the first computer-based programmer offering a detailed explanation of each.

phase and steps of its planning model, which makes the model stronger than several previous social marketing planning approaches^12^. A Social Emotional Learning (SEL) plan was selected for designing interventions based on characteristics of the community.

## Adolescence and social marketing

Social marketing typically is a program-planning process that uses commercial marketing concepts and methods to promote voluntary behavior change. Social marketing promotes the acceptance, rejection, modification, abandonment, or maintenance of specific behaviors by groups of individuals, generally referred to as the target audience^13^. However, social marketing is not a theory in itself, insights are grasped from other bodies of knowledge, like anthropology, psychology, communication theory, and sociology, to affect behaviour^14^.

Social marketers, like their marketing partners, use the four elements of product, price, place, and promotion (4 Ps) to persuade the audience to buy or adopt new behaviors. Using “product” element means designing of an item or an idea and increasing its desirability to the target audience in order to change their behavior. Considering “price” in a social marketing intervention indicates trying to decrease various tangible and intangible costs that a person must accept in order to engage in the special behavior. “Place” stands for channels of distribution for delivering the idea or program. Such as media or influential media and “promotion” denotes strategies and tactics for attracting the target audience and encouraging.

engagement in the behavior, such as incentive^14^. Thus, social marketers increase attractiveness of a behavior, and in some cases, offer products and services to support that behavior. They change cost of one behavior compared to the other behaviors. They make it easier to move towards new behavior, or enhance new behavior by short-term and long-term benefits^15^.

During the process of program design after conducting market research in the study, it was concluded that a Social Emotional Learning (SEL) program can be the best option to promote positive development and subjective well-being of participating adolescents.

## SEL skills

SEL programs are a form of PYD-related developmental assets focusing mainly on positive outcomes including school, life, and career success while also demonstrating evidence regarding effective prevention of negative outcomes. SEL interventions promote personal strengths in youth overlapping considerably with DAP internal assets of social competencies, positive identity, positive values, and commitment to learning^16,17^.

SEL skills are considered to be a combination of social skills. However, they go beyond the limits of social skills and entail developmental process related to understanding and managing one’s relationships with the others. According to the collaborative for academic, social, and emotional learning (CASEL, 2012), there are five essential elements for social and emotional competency including self-awareness (being able to understand one’s own feelings, wishes, strengths, and values, and establishing an optimal sense of self-confidence), self-management(managing and regulating emotion, and supervising one’s own improvement toward achievements, and expressing emotions in a proper way), social awareness(the ability to look at issues from the other’s viewpoint, as well as accepting and respecting both group/individual similarities and differences), relationship skills(the ability to communicate positively and healthily with the others, cooperate with the others and resolve conflicts with the others peacefully), and decision-making(the ability to make responsible decisions according to values and social and ethical standards of the community)^17^.

Studies have also confirmed cost-effectiveness of SEL interventions^18^. SEL skills are essential for the student’s success within and outside the school and are associated not only with proximal gains in the student’s academic performance and decrease in conduct problems but also with the student’s later choices regarding education and employment^19,20^. Adolescents may especially need to develop social and emotional skills as they are learning how to handle new challenges and demands in school and social life while dealing with new, intense emotions (both positive and negative), and they are increasingly realizing that they should do so without adult guidance. SEL programs can help them navigate these difficulties. SEL programs try to help adolescents cope with their difficulties more successfully by improving skills and mindsets^21^.

Researchers have used SEL programs successfully so far. For example, the Strong Kids/Teens curriculum (45 min classes once per week for 12 weeks) designed by Merrell et al., which were conducted among adolescent students showed significant increase of student’s knowledge of healthy social-emotional behavior as well as a decrease of their symptoms of negative affect and emotional distress^22^. Another successful program was the 4R’s Program conducted by Jones et al. 4R’s stands for The Reading, Writing, Respect and Resolution. This school-wide intervention program has three components: (a) a literacy-based curriculum to promote conflict resolution and social-emotional learning, (b) training and improving coaching of teachers in the delivery of the 4Rs curriculum, and (c) a family-based parent–child homework plan^23^.

PATHS (Promote Alternative Thinking Strategies) is another example of the SEL programs targeting self-control, emotional understanding, positive self-esteem, healthy relationships and interpersonal problem-solving skills in children and adolescents. This intervention suggested an improvement in social–emotional competence, pro-social behavior, engagement, and decreased emotional symptoms and peer and conduct problems among students^24^. However these program showed a small effect size^22–24^.

Results of a meta-analysis have also supported the use of SEL interventions in schools to promote healthy development, social and emotional skills, attitudes, behavior, and academic performance^17^.

Among a few studies conducted on evaluation of the effect of SEL programs on adolescents, none of them have investigated the effect of intervention on developmental assets using the developmental asset’s framework and also social marketing planning approach^25–28^. The results of studies conducted in the field of SEL interventions have indicated that design of interventions is better to be based on characteristics of the context and target audience to have optimal effect^29^. Therefore, using social marketing in designing a SEL program in the present study could address this gap and lead us to design the programs tailored to the audience. Accordingly, the present study was done to evaluate effectiveness of SEL intervention designed based on social marketing on developmental assets and well-being of female adolescents in Yazd city.

## Characteristics of the research community

The study population included 190 female adolescent students in Yazd, Iran who attended secondary schools (grades 7–11) and ranging in age from 13 to 18 years (mean 15.42 + /−1.82 years). Most adolescents (82.35%) either had no sibling or had only one. The birth rank of the majority of participants (82.20%) was first or second. A majority of fathers (n = 139, 73.40%) and mothers (n = 145, 76.60%) did not have university education. According to the self-reporting of adolescents, the economic status of the family was mostly (n = 115, 61%) good or very good.

## Materials and methods

The present mixed-method semi-experimental study with two groups (case–control) was performed during 2018–2019. An intervention using social marketing approach was designed and was conducted to the intervention group. The evaluation of the effectiveness of the intervention was carried out in a convergent parallel design. Three Repeated-Measures (pre-test, post-test two months after intervention, and a follow-up six months later) tests were conducted to evaluate effectiveness of intervention. A process evaluation was also conducted within the initial two weeks of the intervention in order to capture opinions of the participants and revise and strengthen the program if needed. A qualitative evaluation was also done to deeply evaluate the achieved results of the interventions.

Data were analyzed with IBM SPSS statistics software and R programming language. Prior to analyses, the data was preprocessed and assumptions were met. At pre-intervention, no significant differences between the intervention and control groups were observed. An independent samples t-test was conducted to determine whether significant differences were observed between the intervention and control groups following the intervention.

A one way repeated measures ANOVA with post hoc analysis with Bonferroni was conducted for to explore the influence of group (intervention or control) as independent variable on the categories of development assets including support, empowerment, boundaries and expectations, constructive use of time, positive values, social competencies, positive identity and commitment to learning as dependent variables. Between groups and within group comparisons was conducted by Tukey and Bonferroni tests, respectively. All tests in these analyses were considered significant at the 0.05 level. There was no extreme outliers in the data.

### CDCynergy social marketing edition

**Figure 2:**
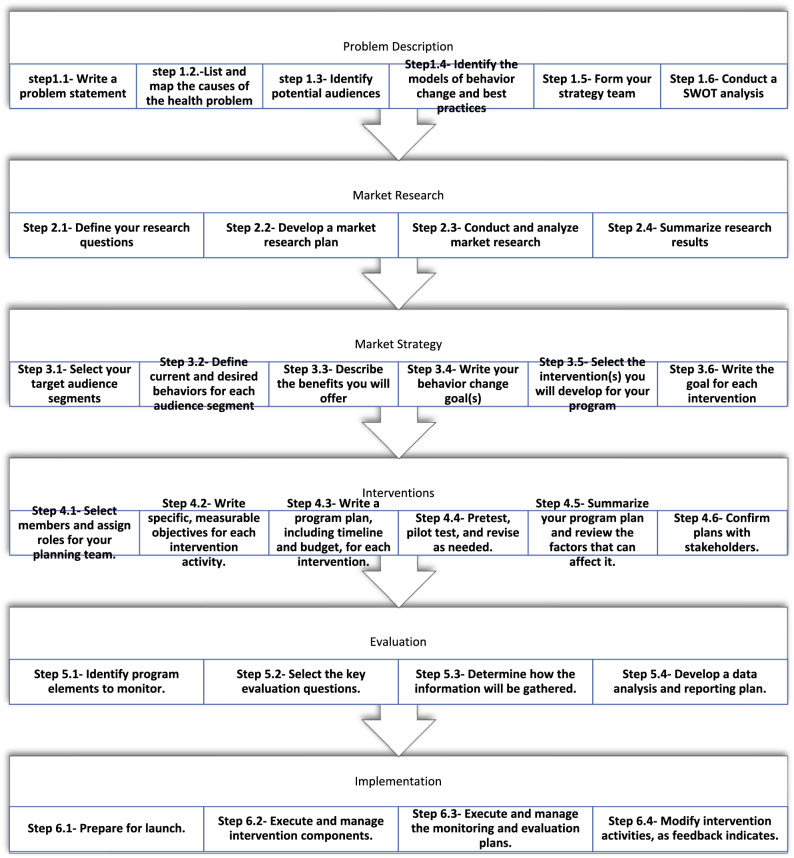
CDCynergy social marketing guide phases and steps.

All stages of this study were planned using CDCynergy social marketing edition online software available at https://www.orau.gov/cdcynergy/soc2web/default.htm. This software has six phases including Problem Description, Market Research, Marketing Strategy, Interventions, Evaluation and Implementation respectively the second phase including market research, is the most important part of the program^30^. All phases and steps of CDCynergy social marketing edition guide (Fig. 2).

### Sampling

Participants were recruited via multi stage sampling. Initially, one first secondary school and one second secondary school for females were selected from each of the two educational districts of Yazd via simple random sampling. Subsequently, one of the two first secondary schools and one of two second secondary schools were considered as the intervention group and the other as the control group by simple random sampling. In the mentioned schools from each 7th, 8th and 9th or 10th, 11th and 12th grade classes, one class were enrolled by random in the study. All students of the classes were included in the study if they met the inclusion criteria including being a student of public secondary schools in Yazd city, being a student of one of the 7–12 grades, having the informed consent of the student and parents and having no history of any mental illness. The exclusion criteria was refusing to continue participating in the research for any reason and being diagnosed with any mental illness.

### Quantitative study

The EPOCH scale was developed by Kern et al., based on the positive emotion, engagement, relationships, meaning, and achievement (PERMA) scale and has 20 items. This scale is a multidimensional scale measuring five positive psychological traits including engagement (the capacity to be involved and focused on what one is doing, as well as participation and interest in life activities and tasks), perseverance (refers to the ability to pursue one's goals until they are accomplished, even in face of obstacles), optimism (referred to as hope and confidence in the future and the desire to take a favorable view of things, and is an explanatory style characterized by evaluation of negative events as transient, external, and situational events), connectedness (refers to the feeling of a person about how satisfying are the relationships with the others and believing that he or she is cared for by the loved, trusted, and valued people, and providing friendship and support for the others),and happiness(means having stable states of positive mood and feeling happy with life, and not having immediate emotions)^31^. The DAP was developed by the Search Institute (2004) and has 58 items measuring 40 developmental assets^32^. The participants were asked to rate the extent to which a phrase has been true for them, at the present moment or in the past three months. The options included not at all or rarely(0), somewhat or sometimes(1), very or often(2), and extremely or almost always(3)^33^. Using the original questionnaires we developed a Persian version of both questionnaires of DAP and EPOCH. Validity and reliability of Persian versions of both questionnaires have been confirmed and reported in another paper by the authors^34^.

We investigated psychometric properties and factor structure of Persian version of DAP and Epoch questionnaire using the confirmatory factor analysis. A sample of 300 female students of the first and second grades in Yazd high schools (other than intervention and control group) were recruited using the multi-stage sampling method. The data were analyzed by Lisrel software. The questionnaire was translated and localized using the standard method. Test re-test method was applied and ICC was calculated as the relative repeatability index to evaluate the questionnaire's reliability. As the Table 1 shows, the ICC for the classes of the DAP questionnaire had an acceptable level and was between 0.80 and 0.96, and for the EPOCH questionnaire it was between 0.68 and 0.85, which indicates the good reliability of the questionnaires (Table 1).

**Table 1:** The intraclass correlation coefficient of categories of DAP and EPOCH (sample size: 573).

Questionnaire	Categories	Number of items	ICC
DAP	Support	7	.91
	Empowerment	6	.85
	Boundaries and Expectations	9	.84
	Constructive use of time	4	.86
	Commitment to Learning	7	.93
	Positive Values	11	.93
	Social Competencies	8	.87
	Positive Identity	6	.80
EPOCH	Engagement	4	.68
	Perseverance	4	.79
	Optimism	4	.85
	Connectedness	4	.85
	Happiness	4	.84

The confirmatory analysis factor was also applied to evaluate the construct validity of the questionnaire. The significance indexes included Root Mean Square Error of Approximation (RMSEA), Chi-square test(× 2, *df*), The goodness of fit index (GFI), confirmatory fit index (CFI), Incremental Fit Index (IFI), and Non-Normed Fit Index (NNFI). We confirmed the existence of boundaries and expectations, support, empowerment, and constructive use of time factors in the external assets as well as positive values, social competencies, positive identities, and commitment to learning factors in the internal assets. The results showed appropriate validity and reliability for the Persian version of both EPOCH the DAP questionnaires. GFI was also confirmed for the factor structure of the questionnaires (Table 2). According to the results of the present study, the Persian version of DAP and EPOCH can be used as an appropriate tool to measure the developmental assets and wellbeing of adolescents in high schools^34^.

**Table 2:** The estimated values of fit indices of the external and internal assets and EPOCH models (sample size: 573).

Fit Indices	Acceptable Rate	Estimated Values		
		Internal Assets	External Assets	EPOCH
X2/df	3 <	2.13	1.72	2.82
GFI	.9 <	.9	.98	.91
AGFI	.9 <	.97	.93	.98
CFI IFI	.9 < .9 <	.92 .92	.92 .92	.97 .92
NFI NNFI	.9 < .9 <	.96 .90	.95 .99	.97 .95
RFI	.9 <	.94	.91	.95
RMSEA	0.08 >	0.059	0.068	0.075
p-value	.05 >	.042	.000	.000
chi square	0.05 <	341.43	5.3.31	1,206.43

### Market research and strategy

In the present study, this phase was performed as an explanatory mixed-method study. The results of market study are important as they provide us with a good understanding of the audience and are used to select appropriate interventions. In Table 3, a summary of results of our market research based on key concepts of social marketing is provided to better understand how we designed interventions and strategies. Intervention strategy and tactics for parents and students based on social marketing principles are presented in Table 3.

**Table 3:** Intervention strategy and tactics for parents and students based on social marketing principles.

TARGET AUDIENCE: For helping this specific target audience: Adolescent girls BEHAVIOR CHANGE: Do this specific behavior: Promoting social competencies and positive identity EXCHANGE/BENEFITS: We will offer the following benefits wanted by the audience: Gain a good sense of themselves, success in relationships and future careers, and satisfy their parents STRATEGY: Lessen these barriers, by addressing the following 'Ps':				
Barrier	Product	Price	Place	Promotion
Improper behaviors of parents Lack of knowledge Insufficient support of family and community Lack of social and communicational skills Improper attitudes Low self-esteem	Skills workshops, booklets, and pamphlets	Try and practice in examples of classroom exercises and in the family context	Face-to-face teaching to students in schools and practice in family contexts	Parental education and support of the adolescents in social and emotional skills
TARGET AUDIENCE: For helping this specific target audience: Parents of the adolescent girls BEHAVIOR CHANGE: Do this specific behavior: Help the adolescents to promote their social competencies and positive identity EXCHANGE/BENEFITS: We will offer the following benefits wanted by the audience: Improving their relationships with their children, helping their children to succeed in the future and life, and gaining a good sense of being a qualified parent STRATEGY: Lessen these barriers, by addressing the following 'Ps':				
Barrier	Product	Price	Place	Promotion
Lack of parenting knowledge Lack of parenting skills Improper attitudes	Skills and knowledge booklets Talking to parents using the phone by the researcher	Practice what they have learned in communicating with their children	Learning by reading booklets in their own homes and communicating with the researcher over the phone	Learning using simple explanations, common examples, and practical methods Education in accordance with religious beliefs and using religious hadiths and the Qur'an

### Quantitative market research

To do our market research, initially, a cross-sectional descriptive study was conducted in order to find out the status of developmental assets and to determine the assets needed more intervention. Further details on the method and results of the studies conducted in quantitative part of market research phase have been provided in separate papers^35^.

Over 573 female secondary-school students in Yazd City, aged between 13 and 18 years old were asked to complete the DAP scale as well as EPOCH well-being questionnaire. Results showed that the assets of constructive use of time, social competencies, and positive identity needed more investigations. Then, the desired behaviors were scored from 1 to 10 according to five criteria including risk, effect, behavioral feasibility, resource feasibility, and political feasibility using CDCynergy prioritization wizard. Thus, social competencies and positive identity were selected as educational priorities due to more behavioral, resource, and political feasibility. Therefore, questions of the qualitative study were determined according to assets of these two categories.

### Qualitative market research

A qualitative research was conducted following a quantitative cross-sectional study to better describe the results. Due to the fact that in the cross-sectional study, social competencies and positive identity was the assets in which participant adolescents were weak and needed more intervention, the researchers intended to get more information in the study community about the factors affecting their assets. Results of this study were used to design effective interventions for the study community.

Students who were voluntarily willing to share their experiences and had a full and informed consent were included in the study. Two focus groups of 13 students in 7th grade and 11 students in 8th grade were formed in the classrooms and 11 individual interviews with 10th grade students were carried out in the school yard.

In the beginning, objectives and questions of the interview as well as time and place of group discussion or interview were explained to all participating students. All the data were collected through voice recording and collection of qualitative data continued until data saturation was achieved meaning that we reached a point when our new interviews produces only previously discovered data and no longer added or changed our findings .

The data collection tool was open and in-depth semi-structured interviews. The guide questions of the focus group were determined based on the findings of the quantitative section and focused on cases that needed more intervention and included the following:

1. Has there ever been a time when you did not feel good about yourself and your life and future?
2. Did you feel that you did not have control over your life and future?
3. Have there been times when you did not make good plans and choices?
4. Tell us about your experiences regarding support from people other than parents (support from neighbors, an adult other than parents) in your life.
5. Have there been times when you failed to deal with problems and failures in the right ways? Have there been times when you have not been able to resolve disputes properly or express your feelings properly?
6. Have there been times when there are bad examples in your friends or people around you or in your society or you feel that these things have a bad effect on you? What have you done?
7. Describe your experiences with healthy activities other than studying such as sports and going to the gym, art and creative work, and religious activities that you do.

After asking general questions about the research items, the interview was guided based on the answers provided. Then, if needed, probing questions such as "Can you explain more?" or "Give an example" were used. In addition to this, more detailed and specific questions were also asked in order to explain the details of the obtained information. The average duration of the focus groups was an hour and a half. All interviews conducted in focus groups were recorded. Interviews and focus group discussions were held with duration of between 60 to 105 min (mean 82.13 ± 15.34 min).

The directed content analysis of Lundman & Graneheim (27) with five steps conducted as follows:

1. All notes and audio of the interviews were typed word by word and handwritten at the end of each interview.
2. The whole text was read several times to gain a general sense.
3. The text was extracted and collected into one text, which formed unit of analysis
4. The condensed meaning units were abstracted and coded. various codes were compared based on differences and similarities and were classified
5. The categories were reviewed and discussed by two researchers and then was edited. Finally the basic meaning of the categories shaped the them

To ensure trustworthiness of the data four criteria of Lincoln and Guba 1. Credibility (to what extent we are confident of the 'truth' of the findings), 2. Transferability (to what extent the findings have applicability in other contexts), 3. Dependability (to what extent the findings are consistent and could be repeated), 4. Confirmability (to what extent our findings are neutral or shaped by the respondents and not researcher bias, motivation, or interest) were observed (28).

Credibility was established via prolonged engagement of the researcher with the students (the researcher attended in schools for several months and was in contact with the participants), negative case analysis and member checking (at the end of interviews the handwritings were reviewed by the research participants). Interviewing process and techniques and codes were reviewed by two researchers and also by another panel of expert qualitative researchers.

Transferability of the data were certified by purposeful sampling to form focused groups sample and ensuring data saturation. Dependability was confirmed by measuring coding accuracy and inter-rater reliability of the research team. Confirmability were verified by having external audits; all the research process and product of the research study were examined by another expert panel who were consisted of external qualitative researchers who were not involved in the research process.

Interviews and data entry were conducted by one researcher. Then, coding was done by two researchers and Cohen's kappa coefficient was used to calculate the degree of agreement between the two coders. The items where the agreement coefficient was higher than 0.9 were accepted, and in cases where the kappa coefficient was lower, the codes were modified and Cohen's kappa coefficient was calculated again. It continued until reaching an agreement above 0.9.

### Intervention

Based on the results of market research and CDCynergy prioritization wizard, researchers concluded that SEL program may be an appropriate design to improve internal assets especially social competencies and positive identity. Educational content was developed based on the principles of SEL. After validation of the educational content, the intervention was performed in the intervention group.

SEL intervention was chosen considering the developmental assets, which gained lower scores among study participants. Interventions were selected according to information of quantitative market research. Interventions were also designed according to information related to the key social marketing components (e.g. barriers and facilitators of desired behavior) obtained from participants in the previously conducted market research.

The programs included eight training sessions for students and an educational booklet for parents. Training for female students and their parents was conducted aimed at improving assets in the categories of positive identity and social competencies. The interventions for the students were done by group discussion, role play, lectures, and questions and answers. Class exercise booklets and educational pamphlets were also distributed among students. Each training session for students, lasted about 35–50 min. Lessons were taught in a group format and they were typically ended up with a discussion about the presented topic. Students were guided through practice exercises. They were also encouraged to participate actively in the class, besides homework was assigned to provide students with the possibility to practice and review skills. Titles and brief descriptions of the educational intervention are provided in Table 4.

**Table 4:** Titles and brief descriptions of the educational interventions.

Target audience	Behavior change goals	Tactics and activities	Titles of educational contents
Students	Increase in positive identity	Creating a positive view on oneself and one’s future Increasing self-esteem Practicing goal-setting skills and achieving the goal	-Identifying and displaying personal strengths -Discovering one’s likes, dislikes, qualities and future goals -To learn about one’s positive qualities -to increase awareness of the physical self; to become aware of the media influence on self-image and behavior -To recognize already developed job skills to understand what goals are and their importance to practice setting short term goals to practice goal-setting by developing a contract with a supportive partner to identify accomplished goals and set long-term goals to look at 10-year life plans and evaluate them to learn a step-by step process for setting and achieving goals to practice applying a goal setting model to a personal goal
	Increase in social competencies	Training about decision-making skills Fighting against bad influences Ways to deal with anger Resolving conflict through negotiation Expressing emotions in the right way	to introduce decision making to learn a model for effective decision making to practice to applying the decision-making model to predict the consequences of certain decisions and how they might affect future life plans to practice resisting influences and following through with decisions introduction to community to define communities based on relationships to define rights and responsibilities and understand that people should not violate other’s right to consider why people do or do not accept other people from different groups To clarify what communication is and what makes it effective To identify positive and negative ways of communication To practice expressing thoughts and feelings through “I statement” To understand the importance of sending clear, accurate messages to learn difference between assertive, aggressive and passive behavior to assess assertiveness to role-play assertive ways that teens can ask for what they want or need to role-play assertive behaviors to refuse requests to explore feelings about relationships to clarify the definitions of family and determine the nature of relationships in family to identify the privileges and responsibilities of family membership to encourage communication between teens and parents to identify the qualities of a good friend to recognize different kinds of friends to explore the limits of friendship to identify the adults to go to for help
Parents	Improving parenting behaviors Promoting positive identity of children		Educating parents about proper parenting Teaching about the fact that parental misconduct destroys adolescents҆ self-esteem Teaching about how to help them set goals and a have positive outlook on the individual future in adolescence Teaching how to increase adolescents҆ self-confidence
	Improving parenting behaviors Promoting social competencies of children		How to teach decision- making by parents to adolescents How to express emotions in the right way in the family How to resolve conflicts with children through negotiation

### Qualitative evaluation

The present qualitative research was conducted to better investigate experiences of participating girls about the effects of the interventions. This study was conducted among the students in the intervention group.

Data collection was started with the following question: "Was there a time when you could benefit from the intervention?" and continued by asking exploration questions, such as “How it happened”, "Could you explain more?", or "Please give an example" with respect to the received answers in order to obtain details. Interviews and focus group discussions were held with duration of between 52 and 84 min (mean 64.92 ± 10.76 min).

All methodology steps and considerations of the qualitative evaluation was similar to the qualitative market research which was done prior to the intervention.

### Ethical approval and consent to participate

The study was according to the guidelines of the Declaration of Helsinki. In this study, informed consent was obtained from a parent and/or legal guardian students. The steps of its implementation were approved by the Ethics Committee of Shahid Sadoughi University of Medical Science and Health Services with the Ethics Code of IR.SSU.SPH.REC.1396.115.

## Results

### Results obtained from process evaluation

Results of process evaluation showed the participants' satisfaction with the content and training sessions and applicability of the interventions. The table below shows the extent to which students agree with items related to process evaluation (Table 5).

**Table 5:** Student’s agreement rate with items of process evaluation (sample size: 90).

Agreed: number (percentage)	Questions
90(92.8)	The training content and sessions are of high quality
86(88.7)	I need the materials and sessions, which are training
77(89.4)	The content and training sessions are interesting
86(88.7)	These tutorials and sessions teach me new information
87(89.7)	These materials and training sessions teach me new skills
85(87.6)	These materials and training sessions try to improve my attitude
83(85.6)	I can use the taught lessons
83(85.6)	I participate in practices in class
92(94.8)	The teacher teaches the skills well
90(92.8)	The teacher interacts well with the students
79(81.4)	In general, I have a positive evaluation of the program
68(70.1)	As a result of these trainings, I intend to…

### Quantitative results

A total of 197 adolescent students in the intervention and control groups participated in the study. From which seven participants were excluded from the study due to invalid pattern of responding or excessive number of blank answers. Normality of the data was assessed using the Kolmogorov–Smirnov test, which showed a normal distribution of the data. Mean age of the participants in the intervention and control groups was 16.15 ± 1.66 and 15.86 ± 1.73 years, respectively.

Results of the Independent-Samples t-test comparing the two groups in terms of demographic characteristics including age, GPA in the last year, grade, number of siblings, birth order, father alive, mother alive, father’s level of education, mother’s level of education and family economic status showed no significant difference between two groups (p > 0.05).

The Table 6 below shows the results of the intervention based on the Repeated-Measures test. As presented in Table 7, the main effect of the intervention on social competencies of the students was significant in both time zones of two and six months after the intervention (*P* = 0.02). The associated ES was on the cusp of “small” using Cohen's (1992) classification^36^. The main effect of the intervention on other variables was not significant (*p* > 0.05).

**Table 6:** The effect of the intervention on developmental assets and well-being constructs based on the results of the Repeated-Measures ANOVA test (sample size: 190).

Variable	Group(n)	Pre	2 months	6 months	SS	F	p	ŋ^2^
Support	Intervention	19.70 ± 8.02	20.84 ± 5.37	20.75 ± 5.77				
	Control	19.53 ± 5.18	20.11 ± 5.81	19.99 ± 5.21	42.85	0.59	0.39	0
Empowerment	Intervention	20.17 ± 4.88	20.76 ± 5.06	20.08 ± 5.07				
	Control	20.07 ± 4.68	20.75 ± 5.42	20.47 ± 5.64	1.05	0.02	0.958	0
Boundaries and expectations	Intervention	20.13 ± 4.86	21.04 ± 4.83	20.40 ± 4.91				
	Control	20.55 ± 4.85	19.68 ± 4.92	19.05 ± 4.93	83.79	1.64	0.226	0
Constructive use of time	Intervention	12.46 ± 6.71	14.52 ± 6.49	14.21 ± 6.95				
	Control	12.79 ± 5.98	13.88 ± 6.56	12.75 ± 6.40	50.01	0.56	0.41	0
Positive identity	Intervention	16.96 ± 5.80	20.76 ± 5.68	21.07 ± 6.11				
	Control	18.34 ± 4.80	19.98 ± 5.11	19.71 ± 5.47	9.25	0.18	0.527	0
Commitment to learning	Intervention	21.18 ± 5.25	21.35 ± 4.18	20.61 ± 4.88				
	Control	20.76 ± 5.08	21.15 ± 5.43	19.81 ± 5.75	32.24	0.61	0.415	0
Social competencies	Intervention	16.66 ± 4.61	20.52 ± 5.66	19.80 ± 5.99				
	Control	17.24 ± 4.74	18.32 ± 4.48	17.83 ± 5.34	205.24	4.89	0.02	0.03
Positive values	Intervention	19.32 ± 5.06	20.99 ± 4.86	20.31 ± 5.15				
	Control	18.90 ± 4.54	20.18 ± 5.12	19.40 ± 4.97	73.41	1.59	0.147	0.01
Engagement	Intervention	34.23 ± 8.29	37.01 ± 7.45	36.34 ± 8.69				
	Control	35.98 ± 7.29	36.67 ± 7.62	36.53 ± 7.39	41.61	0.38	0.226	0
Perseverance	Intervention	39.27 ± 7.36	40.11 ± 7.03	39.67 ± 6.90				
	Control	39.72 ± 6.25	39.14 ± 6.85	38.40 ± 7.44	51.19	0.58	0.925	0
Optimism	Intervention	40.27 ± 8.53	42.10 ± 6.18	42.68 ± 6.48				
	Control	43.05 ± 6.59	42.30 ± 7.50	41.88 ± 7.26	76.34	0.81	0.189	0
Connectedness	Intervention	40.48 ± 8.36	42.51 ± 7.11	42.77 ± 7.39				
	Control	42.76 ± 6.28	41.83 ± 7.56	42.21 ± 7.10	17.1	0.15	0.958	0
Happiness	Intervention	38.33 ± 9.14	40.44 ± 7.99	40.47 ± 9.88				
	Control	41.37 ± 7.31	41.31 ± 7.92	40.62 ± 8.23	265.05	1.77	0.197	0

**Table 7:** Results of pairwise comparisons for changes in social competencies.

comparison			p	Adjusted P
Between groups	Intervention vs control	Pretest	.358	.358
		Posttest 1	.00316	.00316
		Posttest 2	.0126	.0126
Within groups	intervention	Pretest vs posttest1	< .000	< .000
		Pretest vs posttest2	< .000	< .000
		Posttest 1 vs posttest 2	.548	1.00
	control	Pretest vs posttest1	.044	.131
		Pretest vs posttest2	.292	.876
		Posttest 1 vs posttest 2	.311	.933

*Between group; Tukey, **within groups; Bonferroni.

Results of pairwise comparisons for changes in social competencies are presented in Table 7.

### Qualitative results

As can be seen, findings of the qualitative evaluation suggested a positive effect of the intervention on promotion of social competencies and positive identity. Additional reported positive changes not evaluated directly in the questionnaire included improving anger management, communication with family members, improving the ability to say no and defend one’s rights, and expressing desires. A total of 385 interview codes, 167 merged codes, eight subcategories, four categories, and one theme were obtained from the data in this section. The integrated codes obtained from the data in this section are shown in Table 8.

**Table 8:** Categories, subcategories, and code samples obtained from qualitative evaluation (sample size: 48).

Category	Subcategory	Code samples
Positive effect on peaceful conflict resolution	Effectiveness of the contract in achieving the goals	Repeated use of the contract to achieve goals Success in achieving the goal by following the contract Improved scores by contract Learn how to think by contract
	Satisfied with resolving conflict in the right way	Resolving conflict about adolescent’s favorite sport by negotiating with the father Considering the father's wishes regarding the teenager's education Finding win–win solutions to meet one’s own as well as the parents' needs Making a contract with the parents about the tablet Creating a pleasant memory using the contract Agreement with the brother to help him solve the exercises in exchange for having the book
	Failure in developing contracts	The tendency to choose the ones҆ desired option and ignore the wishes of parents, in the stage of brainstorming and writing solutions Insisting on one's own wishes without considering the wishes of the parents Inability to resist against one's desires
Improving planning and decision-making skills		Usefulness of decision-making exercises Visualizing the consequences of decisions Avoiding selection of minor things that are harmful to the teenager's goal Having lessons҆ meetings and clubs together
Improving resistance skills		Keeping to say no in face of the other’s insistence on drinking Resisting with a firm tone against alcohol drinking Expressing resistance by showing dissatisfaction with the other party's insistence on drinking
Improving interpersonal skills	Improving communication by using appropriate phrases	Reducing conflict with the younger sister using “I-initiating statement” Improving the teenager's relationship with the father using “I-initiating statement” Changing the behavior of parents following improvement of adolescents҆ behavior Not complaining by the father when the teenager goes out using “I-initiating statement”
	Using communication bridges and avoiding communication barriers	Not insulting in fights with older sister Avoiding aggression against siblings Not swearing at the younger sister Not hitting the younger sister
	Improving anger management	Reducing aggression in family after training Expressing one’s feelings correctly in times of anger Not hurting the others in times of anger Avoiding expression of the feelings angrily to the mother
	Improving communication with family members	Gaining the ability to express the cause of one’s discomfort to mother Sharing feelings with the others instead of closing the door tightly Mom’s satisfaction with the improved adolescents҆ behavior Gaining more love from mother following improvement of adolescent’s behavior Mothers҆ permission for the adolescent to go to birth party due to the improved adolescent’s behavior Fewer fights with brother
	Ineffectiveness of “I-initiating statement”	Ineffectiveness of “I-initiating statement” at all Incorrect wording of” I-initiating statement” Interference of other sister in fighting with younger sister Starting physical fight Little sister's stubbornness in response to phrases that do not continue with “I-initiating statement” Turning to threatening to get ones҆ desires Using force to get desired results
Improving assertiveness skills	Gaining the skill of saying no	Improving courage against unreasonable demands of the younger sister No longer instructing the teenager regarding treating with younger sister after saying no several times Learning how to say no firmly Gaining the ability to keep saying no Understanding benefits of saying no despite feeling the initial discomfort Saying no instead of behaving aggressively No longer instructing the adolescent regarding treating with little sister
	Gaining the ability to defend one’s rights and express one’s wishes	Expressing empathy instead of silence in times of disagreement Not violating one’s own rights in front of the others with an assertive attitude Improving the teenager's relationship with the mother Following the teenager's assertiveness Gaining the ability to say one’s words to mother Deciding for oneself instead of the mother Reduced expression of anger on the little sister
	Ineffectiveness of assertiveness attempts	Still having problem in saying no Inability to say no to a friend's request Wrong start in responding to a friend's request Fear of making a friend upset
Developing positive identity	Becoming purposeful in life	Learning how to strive for one’s goal Finding the purpose of life from the beginning of the sessions Not choosing small things that are harmful to the teenager's goal Deciding never to be disappointed More studying after training
	Gaining a positive outlook on the individuals҆ future	Gaining positive vision for the future Effectively capturing image of the future Learning to think positively Feeling of getting closer to the future by drawing a picture of the future
	Increasing self-esteem	Increased self-confidence from the beginning of training Gaining the courage to speak in front of the boys in the language-teaching class Increasing self-confidence by not having negative thoughts Increasing self-confidence by not criticizing yourself too much
	Ineffectiveness of planning and goal-setting	Inability to set goals due to receiving no help from the others Not getting goals due to not supporting the adolescent by parents Not getting goals in some cases due to the limitations of society Not getting goals due to excessive parental control Not getting goals due to the lack of freedom in society

## Discussion

The results of the present study suggest that the designed intervention might have small but significant effect on the promotion of asset in the category of social competencies. Social competencies in the developmental assets’ framework consist of five assets including planning and decision-making, interpersonal competence, cultural competence, resistance skills, and peaceful conflict resolution^5^. The results of qualitative evaluation also advocate an increase in social competencies in participating adolescents which might be resulted from the intervention. The study on adolescent girls participating in the present study suggest improvement of planning and decision-making skills, interpersonal competence, resistance skills, and peaceful conflict resolution in these adolescents.

Results of the Repeated-Measures test implied small but significant changes in the assets related to social competencies in the present study, which lasted after six months. Numerous studies on evaluation of SEL programs have achieved similar results for instance; Castro-Olivo (2014) argue that SEL intervention had significant effects on SEL knowledge and social-emotional resiliency^27^. Coelho et al., showed that implementing a Portuguese middle school SEL program increased social awareness and self-control and decreased levels of social anxiety^37^. Bierman et al., displayed significant effects of SEL program on promoting social competence and reducing aggressive behavior problems^38^.

Interventions were expected to have a positive effect on social competencies. Because, relationship skills, expressing emotions in proper ways, peaceful conflict resolution, decision-making, and respecting the individual’s differences are emphasized in the present SEL programs. Moreover, considering that the interventions tried to increase the adolescent's self-confidence, her knowledge of individual abilities, values and feelings, goal setting, and developing plans to achieve goals, positive identity was expected to promote, but changes in positive identity were not significant based on quantitative evaluations. This result may be due to the other factors influencing development of identity, since identity development is a social phenomenon and variant factors, such as environment, socio-economic factors, school, peers, and media also influence the adolescents' identities^39^. Furthermore, studies have shown the effect of context on effectiveness of SEL programs. For example, it has been revealed that school poverty is related to lower effectiveness of the program, which may be due to not proper implementation of the programs in the mentioned schools^29^.

Study results of Duerden et al., unlike the present study, showed the positive effects of their intervention on positive identity in the participant boys and girls. These differences can be due to the difference in the type of interventions implemented. Because in the mentioned study, a two-week adventure recreation program, Camp WILD (Wilderness Instruction and Leadership Development) was carried out. Examining the opinions of the participants in the mentioned study also indicated that the program implemented for teenagers provided the possibility of new experiences challenges, learning and practicing new skills in a recreational and fun environment outside of school^40^. While the present study was limited to the school environment and the possibility of outdoor experiences was not available for the adolescents. Also, Gubitz, and Kutcher, emphasize that providing experientially-based outdoor activities for teenage girls can facilitate the promotion of positive identity in them^41^.

Results of the qualitative study conducted after the intervention to investigate the student’s experiences about the effects of the intervention indicated that the two-way interaction between adolescents and family improved behavior of both parties, suggesting that the parents' behavior towards the adolescent was also changed and improved by promoting the use of “I-initiating statement" as well as other proper communication strategies by the adolescent, and this encouraged the adolescent to use this type of positive and non-threatening expression as much as possible. Adolescents realized that using “I-initiating statement”, in addition to expressing their feelings and desires in proper ways; they can reduce tension and conflict between themselves and their family members, especially as one of their mostly needed desires. This was also considered a suitable exchange for them, according to social marketing principles. This helped them to achieve most of their desires in interaction with their parents.

Two of participants noted their failure to use "I-initiating statement". After conducting in-depth interviews with these individuals, it became clear that infidelity to the taught methods and giving up their attempts may have prevented them from success. They reported that they have stopped their use of proper communicational strategies after facing with initial undesirable reactions of the others and have lost control on their emotions and behaviors in conflict situations. Thus, they have returned to the old behaviors like fighting and insulting or aggression against the other party. Cramer and Castro-Olivo, similarly confirmed that outcomes of SEL programs are moderated by the implementation problems and showed small or no significant effects when implementation problems occurred in adherence to the intervention^17^.

Qualitative findings propose that participating adolescents succeeded in improving communication with parents. Adolescent female students reported that they developed contracts between themselves and their parents. This may helped them to achieve their goals and desires and resolve conflicts, they expressed their satisfaction with learning such an approach and after developing one successful contract, and they were encouraged to repeat their use of contract. Students who had completed their goal setting practice successfully (and delivered their signed contract) (Table 4) reported that they had completed all the training steps required to accomplish the contract. Another approach the adolescents expressed as useful was negotiation. Most of the conflicts in adolescent’s life occurred between them and their parents. As a result, progress in resolving such conflicts with negotiation was significant for adolescents.

The post-intervention qualitative study also revealed the possibility of promotion of the adolescent’s assertiveness in defending their rights. However, in the DAP, regarding the questions only investigating the adolescent’s behavior on staying away from risky behaviors, such as consumption of alcohol, drugs, and smoking as well as resistance against bad influences, during close interaction between the researcher and the adolescents participating in the study, it was found that a majority of the adolescents participating in the intervention suffered from a lack of assertiveness skills in expressing their needs and desires, even in front of their parents. Some of participants, who lacked assertiveness, during the qualitative evaluation, expressed their satisfaction with their successes in the skill of saying no and expressing their wishes directly and in the right way to their parents and the others. They also noted a change in the behavior of the others and an increase in the amount of respect received from the others and the sense of self-esteem after their firm behaviors. Two students reported failing to say no, and after an in-depth interview, it was found that had not followed the steps properly. Given the poor skill of saying no and the assertiveness to express demands in the adolescent participant, it seems necessary to include this issue in the list of new priorities for the future interventions.

According to qualitative findings, the intervention possibly promoted positive identity of the adolescents, helping them in goal setting, teaching the steps of proper planning, and achieving the goals. Participants expressed satisfaction with practicality of the planning process and the effect of a successful sample lecture in class. Participants reported that drawing pictures of their imaginations about the future, implemented in a fun and entertaining way in the classroom, “brought them closer to the future and helped them to gain a positive view of the future”. Also, listening to the life story of successful people in this field “made them to believe in success”. Moreover, using positive expressions and thoughts about oneself and avoiding self-criticism were mentioned as ways that promoted self-esteem in them.

The results of the present study should be interpreted with caution due to limitations of the study including exclusion of incomplete questionnaires from analyses, small sample size, reliance on solely self-report measures, and excluding adolescent boys in the study. In the present study, by assuring the students about confidentiality of the information and asking them to observe truthfulness in self-reporting, we tried to minimized the impact of the limitations of self-reporting on the validity and accuracy of the findings. However, studies using direct assessment of social emotional skills in adolescents is needed to provide more compelling evidence of student stablished skills. Future studies can also use third party data to shed light on social emotional skills and behavior of adolescents in Iran. For promoting PYD in the adolescents, interventions should be performed over a longer period of time and in a wider range to create the desired effect on all of eight categories of support, empowerment, boundaries and expectations, constructive use of time, commitment to learning, positive values, social competencies, encompassing variant contexts of personal, social, family, school and community.

Besides, during this study, after recognizing the audience and interacting with them for a long time, it was found that the ability to express desires boldly is weak among the Iranian female adolescents. This ability has not been investigated in the DAP. This may be due to the great difference between the Western societies where the tool has been developed, which are individualistic compared to the present collectivist society. In Iran, female adolescents refuse to express their desires in order to satisfy parents or important others, causing them to experience great dissatisfaction and inner bad feelings. Therefore, it is suggested to consider the potential of assertiveness in asking for demands from relatives as an additional asset in the developmental asset’s framework.

In sum, the results of the present study suggests a small effect of the social emotional intervention on improving the social competencies of adolescent girls. The smallness of these effects notify that the interventions should be developed more widely, especially by creating opportunities for experience, challenges, social participation and interaction in the environment outside of school and home. For the design of interventions in less restricted environments, plans such as after school programs, can provide a good possibility for the design of innovative interventions.

## Conclusion

Based on the results of the present study, the SEL program might have significant but small positive effect on social competencies of the participated adolescents however; it did not show quantitative significant changes in positive identity as well as other developmental asset’s categories including support, empowerment, boundaries and expectations, constructive use of time, commitment to learning and positive values as well as subjective well-being. It should be noted that changes in other categories of the developmental assets framework were not considered as goals of the present study due to the comprehension of the developmental assets framework and our limited time and resources.

The mixed-method evaluation of the SEL intervention conducted in the present study suggested a mutual nature of effectiveness of SEL as well as hinting adherence and failure to complete the trained steps as being a possible reason to lower success in SEL. In addition, qualitative evaluation added further explanation about not quantitatively measured changes caused by the program.

## Data Availability

The Data which support the findings of the present study is available at the links below: https://drive.google.com/drive/folders/1adJXrUGltTiRVEeab_Bh_VsBSI6LsQx0.
